# Microbe-Metabolite Associations Linked to the Rebounding Murine Gut Microbiome Postcolonization with Vancomycin-Resistant Enterococcus faecium

**DOI:** 10.1128/mSystems.00452-20

**Published:** 2020-08-18

**Authors:** Andre Mu, Glen P. Carter, Lucy Li, Nicole S. Isles, Alison F. Vrbanac, James T. Morton, Alan K. Jarmusch, David P. De Souza, Vinod K. Narayana, Komal Kanojia, Brunda Nijagal, Malcolm J. McConville, Rob Knight, Benjamin P. Howden, Timothy P. Stinear

**Affiliations:** a Department of Microbiology and Immunology, Peter Doherty Institute for Infection and Immunity, University of Melbourne, Melbourne, Australia; b Doherty Applied Microbial Genomics, Department of Microbiology and Immunology, Peter Doherty Institute for Infection and Immunity, Melbourne, Australia; c Microbiological Diagnostic Unit Public Health Laboratory, Peter Doherty Institute for Infection and Immunity, University of Melbourne, Melbourne, Australia; d Department of Pediatrics, University of California San Diego, La Jolla, California, USA; e Department of Computer Science & Engineering, University of California San Diego, La Jolla, California, USA; f Flatiron Institute, Centre for Computational Biology, New York, New York, USA; g Skaggs School of Pharmacy and Pharmaceutical Sciences, University of California San Diego, USA; h Collaborative Mass Spectrometry Innovation Center, Skaggs School of Pharmacy and Pharmaceutical Sciences, University of California San Diego, La Jolla, California, USA; i Metabolomics Australia, Bio21 Institute of Molecular Science and Biotechnology, University of Melbourne, Melbourne, Australia; j Centre for Biostatistics and Clinical Trials, Peter MacCallum Cancer Centre, Melbourne, Victoria, Australia; k Department of Biochemistry and Molecular Biology, University of Melbourne, Melbourne, Australia; l Department of Bioengineering, University of California San Diego, La Jolla, California, USA; m Center for Microbiome Innovation, University of California San Diego, La Jolla, California, USA; Max Planck Institute for Marine Microbiology

**Keywords:** microbiome, multiomics, metagenomics, metabolomics, gut microbiome, vancomycin-resistant enterococci, colonization, antimicrobial resistance, ceftriaxone

## Abstract

Vancomycin-resistant Enterococcus faecium (VREfm) is an emerging antibiotic-resistant pathogen. Strain-level investigations are beginning to reveal the molecular mechanisms used by VREfm to colonize regions of the human bowel. However, the role of commensal bacteria during VREfm colonization, in particular following antibiotic treatment, remains largely unknown. We employed amplicon 16S rRNA gene sequencing and metabolomics in a murine model system to try and investigate functional roles of the gut microbiome during VREfm colonization. First-order taxonomic shifts between *Bacteroidetes* and *Tenericutes* within the gut microbial community composition were detected both in response to pretreatment using ceftriaxone and to subsequent VREfm challenge. Using neural networking approaches to find cooccurrence profiles of bacteria and metabolites, we detected key metabolome features associated with butyric acid during and after VREfm colonization. These metabolite features were associated with *Bacteroides*, indicative of a transition toward a preantibiotic naive microbiome. This study shows the impacts of antibiotics on the gut ecosystem and the progression of the microbiome in response to colonization with VREfm. Our results offer insights toward identifying potential nonantibiotic alternatives to eliminate VREfm through metabolic reengineering to preferentially select for *Bacteroides*.

**IMPORTANCE** This study demonstrates the importance and power of linking bacterial composition profiling with metabolomics to find the interactions between commensal gut bacteria and a specific pathogen. Knowledge from this research will inform gut microbiome engineering strategies, with the aim of translating observations from animal models to human-relevant therapeutic applications.

## Abstract (precis)

This study demonstrates the importance and power of linking bacterial composition profiling with metabolomics to find the interactions between commensal gut bacteria and a specific pathogen. Knowledge from this research will inform gut microbiome engineering strategies, with the aim of translating observations from animal models to human-relevant therapeutic applications.

## INTRODUCTION

Vancomycin-resistant Enterococcus faecium (VREfm) is a significant health care-associated pathogen. VREfm infections can be difficult to treat due to their intrinsic and acquired resistance to nearly all classes of antibiotics (1). The World Health Organization categorizes VREfm as a “high priority” bacterial pathogen, advocating research to stop the global increase in antibiotic resistance (2). Recent studies highlight the importance of the gut microbiota in modulating the growth and virulence of VREfm in the gastrointestinal ecosystem. For instance, the depletion of normal gut flora using antibiotics exacerbates the severity of VREfm infection (3), whereas transplant of commensal species, including a consortium of Clostridium bolteae, Blautia producta, Blautia sartorii, and Parabacteroides distasonis (4, 5) can drive established VREfm colonization to below levels of culture detection. Specifically, B. producta—a colonizer of the colon—reduces VREfm growth *in vivo* by secreting a lantibiotic (6). These observations raise the intriguing possibility that metabolic traits act in concert between pathogen and select gut commensals to confer mutual benefits during pathogen persistence. These findings also highlight the greater risk posed to immunocompromised patients when colonized with VREfm. For instance, allogeneic hematopoietic cell transplantation patients have gastrointestinal tracts that are dominated by VREfm as a result of losing a large portion of the intestinal commensal microbiota upon receiving broad-spectrum antibiotics as pretreatment (7). Hildebrand et al. discovered long-term ecological impacts to the gut microbiome, with strong bacterial species turnover, after ceftriaxone treatment in humans (8). Further, mice receiving broad-spectrum antibiotics (combination of metronidazole, neomycin, and vancomycin) showed markedly increased VREfm colonization of the cecum and colon. The compromised intestinal innate immune defenses in these animals allowed proliferation of VREfm caused by the antibiotic exposure and subsequently reduced the expression of antimicrobial molecules produced by bacteria in the intestinal mucosa (9).

The problem with VREfm is further complicated by the fact that enterococci are members of the gastrointestinal tract microbiota, a key reservoir of antimicrobial resistance (AMR) genes, and potentially facilitating gene transfer within the gut microbiome (10). For example, the *vanB* resistance gene was detected in human fecal specimens that did not contain culturable VRE, and instead, demonstrated that isolates carrying the resistance transposon are anaerobic commensal bacteria, Eggerthella lenta and Clostridium innocuum (11). Colonization of, and persistence in, the gastrointestinal tract therefore presents as a key mechanism for *de novo* VRE and may lead to severe invasive disease.

The current study aimed to understand the impact of antibiotics on the murine gut microbiota and the subsequent colonization pattern of VREfm. To this extent, we designed a murine model time-series study that consisted of two main perturbative phases: (i) antibiotic pretreatment with ceftriaxone and (ii) VREfm challenge. Our 16S rRNA gene profiling analyses highlighted a first-order shift in bacterial biodiversity composition across time, a second-order clustering of samples associated with the experimental phases, and the transition of the post-VREfm colonization gut microbiota and its metabolome toward resembling an asymptomatic carriage-like microbiome phenotype. This research provides support for engineering the metabolic potential of the gut microbiome using, for example, prebiotics as a nonantibiotic alternative for treating multidrug-resistant bacterial infections.

## RESULTS

### Experimental design.

The following experimental design was developed to address the hypothesis that there are specific murine gut microbiome factors that facilitate VREfm colonization; three groups of three C57BL/6 mice (cocaged wild-type males) were monitored and fecal samples were collected over a 14-day period with two intervention time points including (i) ceftriaxone treatment administered at 0.5g/liter in drinking water across a 2-day period, and (ii) colonization (via oral gavage) with 1 × 10^6^ VREfm ST796 per mouse postantibiotic treatment at a single time point. Mice were housed in groups of five, and samples were collected from the same three mice to represent technical replicates per cage; herein, each group of cohoused mice will be referred to as group A, group B, and group C. The remaining two mice per group were reserved for microbiological assays. Table 1 highlights samples and data sets collected.

**TABLE 1:** Summary of samples analyzed in this study

Day of expt	Phase of expt^*a*^	Amplicon 16S rRNA gene data^*b*^	Metabolomics^*b*^	Avg no. of observed sOTUs^*c*^
0	N	✓	✓	210
1	N	✓	−	220
2	N	✓	−	220
3	N	−	−	−
4	N	−	−	−
5	N	✓	✓	225
6	Abx-Tx	✓	✓	112
7	Abx-Tx	✓	✓	84
8	Abx-Wn	✓	✓	93
9	VRE-E	✓	✓	30
10	VRE-E	−	−	−
11	VRE-E	−	−	−
12	VRE-E	✓	−	22
13	VRE-L	✓	−	34
14	VRE-L	✓	✓	53

a The key phases of the experiment where N represents naive, Abx-Tx represents antibiotic treatment, Abx-Wn represents antibiotic weaning, VRE-E represents early-phase post-VREfm colonization, and VRE-L represents late-phase post-VREfm colonization.

b Symbols: ✓, sample processed; −, data unavailable.

c The average number of sOTUs observed across all mice for each day of the experiment.

### Amplicon 16S rRNA gene sequencing revealed first-order shifts in bacterial community composition.

Amplicon 16S rRNA gene sequencing was performed to capture the bacterial community composition in an effort to track changes in response to antibiotic pretreatment and VREfm colonization. Bacterial community profiles were assessed in fecal samples from nine mice before, during, and after the two interventions (Table 1). A total of 71.32% of reads (10,519,073 reads) passed quality control, with 321,955 reads on average per sample and a total of 3,574 exact variant sequence types (i.e., features), with an average of 118 features per sample, and an upper bound of 246 features (when rarefied to 20,000 reads). Alpha rarefaction analysis demonstrated sufficient sequencing depth to capture microbial diversity to saturation (see Fig. S1 in the supplemental material).

10.1128/mSystems.00452-20.1 FIG S1 Alpha rarefaction plot of all samples rarefied to 20,000 reads per sample. Download FIG S1, PDF file, 3.5 MB. Copyright © 2020 Mu et al. 2020 Mu et al. This content is distributed under the terms of the Creative Commons Attribution 4.0 International license.

**FIG 1:**
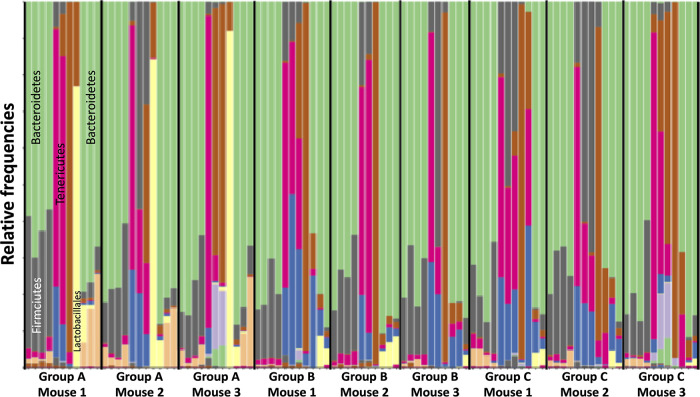
Biodiversity plot of sOTUs as relative frequencies at the taxonomic level of class. First-order shifts in microbial community composition, as revealed by 16S rRNA gene community profiling, from a predominance of *Bacteroidetes* to *Tenericutes* and return to *Bacteroidetes* was observed. Each column displays the relative bacterial community composition in a mouse fecal sample collected daily and sorted by the chronology of the experiment (i.e., day of experiment; Table 1). The columns are further sorted by group (i.e., group A, group B, and group C) and individual mice within each group (mouse 1, mouse 2, and mouse 3). Stacked bars are presented as relative frequencies at the taxonomical level of class, however, annotations of key taxa are at the phylum level (*Bacteroidetes* [green], *Firmicutes* [gray], and *Tenericutes* [fuschia]) or order level (*Lactobacillales* [yellow]).

The biodiversity profiles of each sample were compared and showed that key sub-operational taxonomic units (sOTUs) were differentially abundant throughout the course of the experiment (Fig. 1). There was a shift in the dominance of *Bacteroidia* (*Bacteroidetes*; light green colored bars) during the naive phase of the experiment to *Mollicutes* (*Tenericutes*; fuschia colored bars) in response to ceftriaxone treatment, with a return to the predominance of *Bacteroidia* during the late phase of the experiment, after VREfm colonization (i.e., days 12 to 14). Of note is the predominance of *Lactobacillales* in mouse 1 to 3 from group A (Fig. 1).

### The murine gut microbiota responds to antibiotics, and microbial community richness begins to rebound 3 days after VRE colonization.

**FIG 2:**
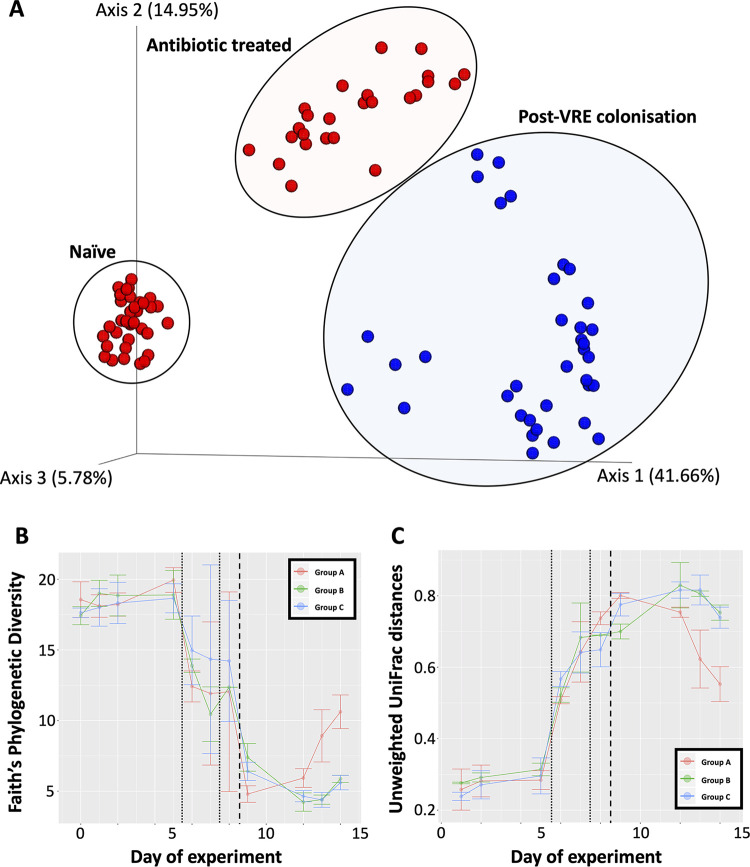
Diversity analyses. (A) Principal-coordinate analysis plot of unweighted UniFrac distances. Data points are projected onto the sample space and colored by pre-VREfm colonization (red), and post-VREfm colonization (blue). Note that circles and ellipses function to highlight the separation of experimental phases and do not indicate statistical confidence intervals. Principal coordinate axis 1 explains 41.66% of the variation observed between the naive microbiota and those from the post-VREfm colonization phase. (B) Community richness of the murine gut microbiome, as measured by Faith’s phylogenetic diversity, in response to ceftriaxone treatment and challenge with VREfm; (C) Community dissimilarity distances, as calculated by unweighted UniFrac, of each time point relative to day 0 (naive phase).

Principal-coordinate analysis (PCoA) of the unweighted UniFrac (12) distances was used to assess clustering of fecal samples based on bacterial composition. This assessment showed that the fecal microbiota from samples collected from each phase clustered together but were clearly separated between phases, after exposure to ceftriaxone, and challenge with VREfm postantibiotic treatment (Fig. 2A). Permutation-based statistical testing demonstrates the groups are significantly different from one another (Fig. S2). Temporal tracking of the changing microbiomes against each mouse on the PCoA sample space demonstrated a clear, unidirectional trajectory that followed the chronology of the experiment (https://doi.org/10.6084/m9.figshare.12775859). Procrustes analyses of weighted and unweighted UniFrac distances showed that the same general patterns on the sample space were preserved, meaning that there is congruency in global spatial patterns between qualitative and quantitative measures of community dissimilarity (Fig. S3).

10.1128/mSystems.00452-20.2 FIG S2 PERMANOVA testing (999 permutations) on unweighted UniFrac distances (16S rRNA gene data) relative to samples from the naive phase. *P* values for pairwise PERMANOVA testing are given in parentheses for the following phases of the experiment: naive and antibiotic treatment (0.001), naive and antibiotic treatment (0.001), naive and infection (0.001), and antibiotic treatment and antibiotic wean (0.025). Download FIG S2, PDF file, 0.3 MB. Copyright © 2020 Mu et al. 2020 Mu et al. This content is distributed under the terms of the Creative Commons Attribution 4.0 International license.

10.1128/mSystems.00452-20.3 FIG S3 Emperor plot of procrustes analysis of unweighted (blue) and weighted (red) UniFrac distance matrices. Download FIG S3, PDF file, 1.3 MB. Copyright © 2020 Mu et al. 2020 Mu et al. This content is distributed under the terms of the Creative Commons Attribution 4.0 International license.

Analysis of community diversity (Faith’s phylogenetic diversity index) revealed a stable and rich microbial community during the naive phase preceding a sharp decrease following antibiotic treatment and a further decrease immediately following VREfm colonization (Fig. 2B). Of note is the responsiveness of the microbiota (within 24 h) to the removal of antibiotics at the end of day 7. Community richness began to rebound at approximately 3 days after VREfm colonization (i.e., day 12), with group A demonstrating a higher rate of rebound compared to groups B and C. Calculating the distances of dissimilarity (unweighted UniFrac distances) of each mouse microbiota time point relative to day 0 (a proxy for the naive bacterial community phenotype) revealed a small dissimilarity distance for samples collected during the naive phase and an increasing dissimilarity distance following antibiotic treatment (day 6) and VREfm colonization (day 9; Fig. 2C). There was a downward trajectory in distance scores 3 days after VREfm colonization (i.e., day 12); group A followed a sharper return to a microbiota resembling day 0. These observations suggest that mice were transitioning toward a persistent carrier-like state, and that the rebounding community richness toward levels representative of the naive phase was by a microbial community structure that resembled the naive phase. Additional studies where the time frame of post-VREfm challenge extends beyond 1 week of monitoring are needed to understand whether the perturbed microbiome will return to resemble an absolute naive state or arrive at a new, altered state.

### Multinomial regression identifies sOTUs most positively associated with VREfm colonization.

**FIG 3:**
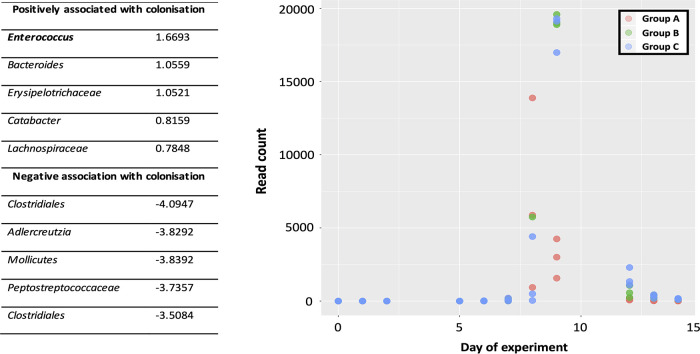
Multinomial regression. Multinomial regression identified an *Enterococcus* exact sequence variant as the most positively associated with the colonization phase (log fold change score of 1.6693). Read counts for the *Enterococcus* ESV tracked daily across the experiment showing high abundance during the days of VREfm challenge.

Multinomial regression using *Songbird* was employed to identify sOTUs that were most positively and negatively associated with the post-VREfm colonization phase (Fig. 3). The five most positively associated sOTUs were *Enterococcus*, *Bacteroides*, *Erysipelotrichaceae*, *Catabacter*, and *Lachnospiraceae*, while the five most negatively associated were *Clostridiales*, *Adlercreutzia*, *Mollicutes*, *Peptostreptococcaceae*, and *Clostridiales*. Temporal tracking of exact sequence variants (ESVs) demonstrated that the ESV feature classified as *Enterococcus*—and identified as the most positively associated with the post-VREfm colonization phase—was most abundant on days of challenge, confirming that this ESV likely was the ST796 VREfm colonization challenge organisms (Fig. 3). There were a further eight ESV features classified as *Enterococcus*; however, they were absent during the days representing VREfm colonization and lacked positive associations with the post-VREfm colonization phase, suggesting that these features represent murine gut commensal enterococci.

### Molecular networking identifies differential metabolome profiles.

**FIG 4:**
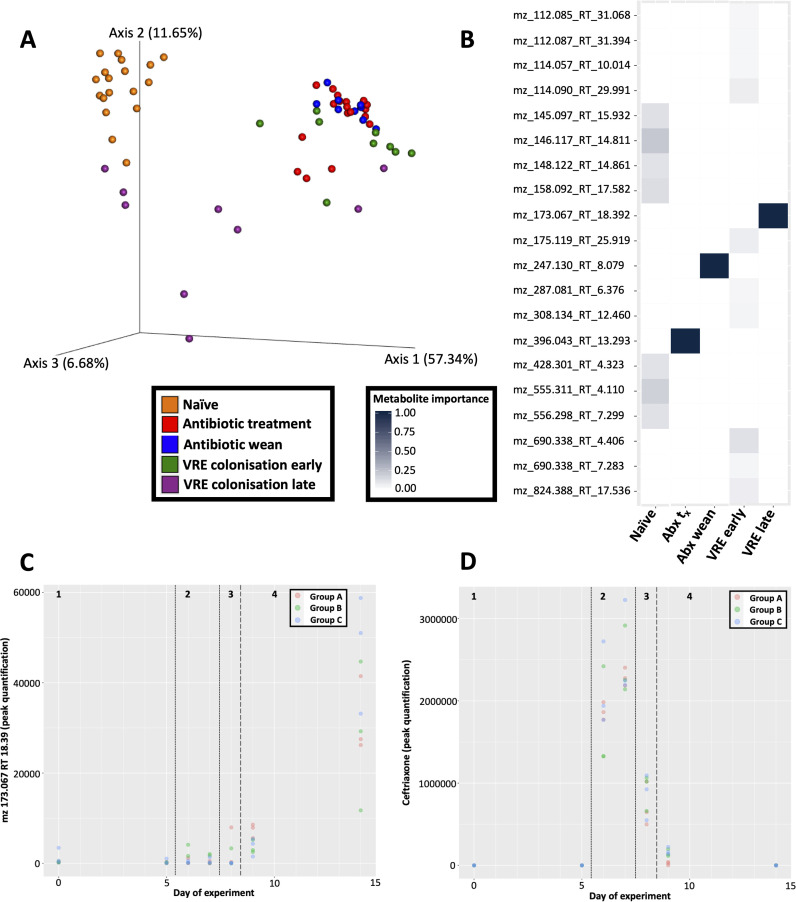
Metabolomic analyses. (A) Emperor plot displaying principal-coordinate analysis of binary Jaccard distances of metabolomic profiles. Samples are color coded, and the colors represent the naive (orange), antibiotic treatment (red), antibiotic weaning (blue), early VRE colonization (green), and late VREfm colonization (purple) phases. (B) Random Forest classifier identifying metabolite features (spectra) for each phase of the experiment. The heatmap is color coded from low ranking score (white; i.e., lowest importance) to high ranking score (dark blue; highest importance). Metabolite features are labeled by their mass-charge ratios and retention times for the reason that current databases do not capture their chemical structure and/or identifications. Abx t_x_, antibiotic treatment. (C) Peak quantification values for feature 6325 (*m/z* = 172.0671 and RT = 18.39) present in abundance during VRE colonization late (phase 4). (D) Peak quantification values for ceftriaxone (*m/z* = 555.0537 and RT = 13.30) tracked across the experiment. Ceftriaxone values are highest during antibiotic treatment (phase 2) and begins to wane during antibiotic weaning (phase 3).

Duplicate fecal samples from key time points throughout the experiment (i.e., days 0, 5, 6, 7, 8, 9, and 14) were analyzed by data-dependent tandem mass spectrometry (MS/MS) performed on a liquid chromatography quadrupole time of flight (LC-QTOF) system to monitor changes in the murine gut metabolome (Table 1). Polar metabolite analysis was given preference in an effort to broadly capture primary metabolites that play a key role in “metabolic hand-offs” that define interspecies interactions. Analysis of the global metabolome profile of each sample was measured based on their overlapping molecules and a PCoA plot using a binary Jaccard distance metric through the Global Natural Products Social Molecular Networking (GNPS) platform (13). A separation of metabolite profiles along PCoA1 was observed (57.34%; Fig. 4A). Metabolomes from the naive and late VREfm colonization phase tended to cluster together, while samples from the postantibiotic phases including the early VREfm colonization phase clustered together. Supporting pairwise permutational multivariate analysis of variance (PERMANOVA) testing (Fig. S4) highlights that naive and early VRE samples are significantly different, while late VRE has a lower distance to naive samples compared to antibiotic-treated, antibiotic wean, and early VRE samples.

10.1128/mSystems.00452-20.4 FIG S4 Pairwise PERMANOVA testing (999 permutations) on binary Jaccard distances (metabolome data) relative to samples from the naive phase. While naive and late VRE samples are significantly different, late VRE has a lower distance to naive compared to Abx txt, Abx wean, and early VRE samples. Download FIG S4, PDF file, 0.2 MB. Copyright © 2020 Mu et al. 2020 Mu et al. This content is distributed under the terms of the Creative Commons Attribution 4.0 International license.

Random forest analysis of spectrum profiles from LC-MS/MS was used to predict experimental phase and rank the importance of metabolite association with each experimental phase. The top metabolite features for each experimental phase are highlighted in Fig. 4B. Unique profiles of metabolite features were observed for each phase of the experiment. Importantly, the late VREfm colonization phase captures an unknown metabolite (feature 6325) with a mass-to-charge ratio (*m/z*) of 173.067 and retention time (RT) of 18.392; this metabolite is exclusively present during what represents the transition toward resembling the naive microbiome. Manual curation of feature 6325 in positive-ion mode predicts a molecular formula C_5_H_8_N_4_O_3_ with ≥10 ppm in mass error. The major peaks in the MS/MS spectrum for feature 6325 are 173.07 (precursor ion, [M+H]+ assumed), 155.06 (precursor ion, 18 [H_2_O]), 113.05 (155.06 product ion, 42.01 [likely C_2_H_2_O]), 43.03 ([C_2_H_2_O+H] plus product ion, further supporting neutral loss of C_2_H_2_O); given the summation of results, the chemical structure of feature 6325 is likely to contain a N-acetylated hydroxyl group. Peak quantification values indicate its presence during the late phase of VRE colonization (Fig. 4C). Further manual curation of MS/MS data identified ceftriaxone as feature 3901 with an *m/z* of 555.0537 and RT of 13.30, and mostly abundant during days of antibiotic exposure (Fig. 4D).

### *Bacteroidales*-associated metabolites implicated in late-phase post-VREfm colonization.

**FIG 5:**
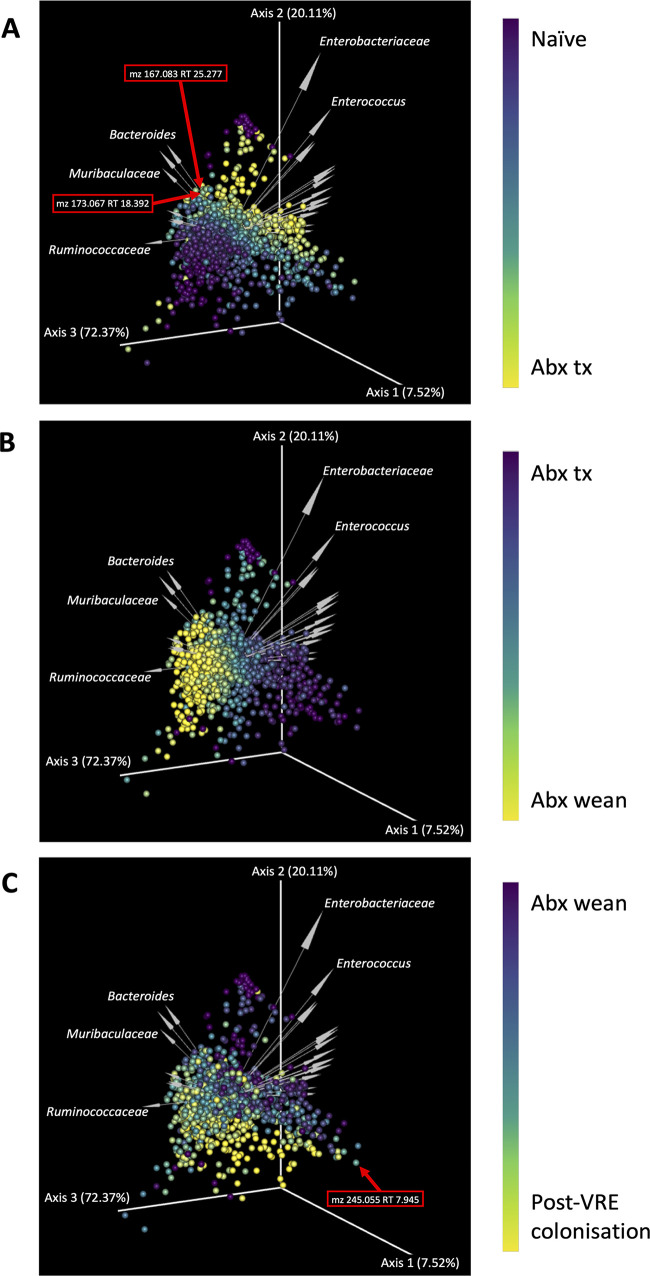
Microbe-metabolite vector biplots. Sequential biplots highlighting the changing metabolite differentials across each key phase of the experiment; Abx tx is the antibiotic treatment phase, and Abx wean is the period when antibiotics were removed for a 24-h period prior to colonization with VREfm. Each point on the sample space represents metabolites, and arrows represent microbes. Microbe and metabolite features are fixed upon the sample space, with gradient coloring of metabolites indicating the transition across key phases of the experiment. The distance between each point is indicative of metabolite cooccurrence frequency, and the angle between arrows indicates microbial cooccurrence. The directionality of the arrows describes the variation in the metabolites explained by the microbes represented by the arrows. For example, metabolite feature 6325 (*m/z* 173.067 and RT 18.392) is demonstrated to cooccur with *Bacteroides*. Information about the abundances of these cooccurring features are provided as heatmaps in Fig. S6 in the supplemental material.

A distinct profile shift in microbe and metabolite abundances (as calculated by multinomial regression) was observed, particularly during late-phase VREfm colonization (Fig. S6). Shallow neural networking analysis with *mmvec* was used to predict microbe-metabolite interactions through their cooccurrence probabilities (Fig. 5). Sequential biplots captured the shift in experimental phases and highlighted the cooccurrences of microbiota and metabolomic data sets (Fig. 5A to C). There was a strong *Enterococcus* effect as indicated by the magnitude of the corresponding arrow, and the rebounding species during the late-phase VREfm colonization are predominantly *Bacteroidales* sOTUs (Fig. 5C) with cooccurring metabolite features *m/z* 173.067 RT 18.392, and *m/z* 167.083 RT 25.277. Metabolite feature *m*/*z* 245.055 RT 7.945 was ranked as being highly associated with the post-VRE colonization phase. These results integrate microbial and metabolite data sets to reveal which microbes may be responsible for detected metabolites. In this instance, the metabolite present during the phase representing a transition toward a microbiome approximating the naive state (feature 6325 *m/z* 173.067 and RT 18.392; Fig. 4B) is linked with *Bacteroidales* (Fig. 5A).

## DISCUSSION

In this study of the murine gut ecosystem, we employed a mouse model of gastrointestinal tract colonization that replicates the shift in bacterial composition when patients enter the health care system, develop an imbalance in their microbiome as a result of pretreatment (e.g., antibiotic treatment), and are subsequently colonized with a hospital superbug (14). The resolution of current studies describes a consortium of commensal microbes that can, for example, reduce the magnitude of VREfm colonization (4, 6); however, understanding the key metabolic shifts relative to the gut microbiota remains challenging (15). Here, we employed amplicon 16S rRNA gene sequencing and high-resolution mass spectrometry metabolomics in an effort toward determining microbiota-metabolome interactions during VREfm colonization. We demonstrated clear changes in the gut microbiome in response to ceftriaxone and VREfm challenge.

Conceptual and statistical advances in analysis of amplicon 16S rRNA gene data (16, 17) whereby OTUs are clustered at a 99% nucleotide similarity threshold allows for the identification of exact sequence variants (ESVs). Query against an error-corrected database (17) can detect multiple ESVs that may be classified to the same taxonomic rank. For example, our analyses identified multiple ESVs classified as enterococci; however, when the relative abundances were tracked across the chronology of the experiment, only one *Enterococcus* ESV was dominant in relative abundances and most positively associated with the days of post-VREfm challenge (Fig. 3). This highlights the resolving power to differentiate between commensal and pathogenic strains of enterococci when the composition of the microbial community is considered. The fact that this was achievable at the level of amplicon 16S rRNA gene sequencing alludes to the possibility of implementing microbiota screenings as routine diagnostics for patients entering health care systems. Further, first-order level shifts in microbial community composition was observed in response to ceftriaxone and subsequent VREfm challenge (Fig. 1). Three days after VREfm colonization (i.e., day 12), the microbiome richness begins to rebound, suggesting that mice are transitioning toward a persistent carrier-like state. Interestingly, the group A cohort exhibited a higher rate of rebound that may be facilitated by their initially higher microbial community richness and predominance of *Lactobacillales* on the day of VREfm challenge (Fig. 2B); this observation supports the need to prescreen “baseline” microbiota profiles of patients upon admission into hospital for the reason that it is not necessarily which microbial populations are removed postperturbation (e.g., antibiotic pretreatment) but instead, which populations persist that drives the responding phenotype. We can begin to assess patients from across different wards (e.g., intensive care unit, oncology, neurology, and healthy cohorts) and build a database of microbiome profiles that can be used as biomarkers to predict: (i) the susceptibility of patients to develop persistent bacterial colonization and (ii) propensity to clear the pathogen once colonized. The clinical implication is that new patients are screened and identified (via beta-diversity meta-analyses) by these biomarkers and placed in bedding cohorts accordingly, thereby improving infectious disease management and isolation precautions within health care-associated ecosystems.

The shortlist of microbes ranked as most negatively associated with the colonization phase (*Clostridiales*, *Adlercreutzia*, *Mollicutes*, *Peptostreptococcaceae*, and *Clostridiales*; Fig. 3) are hypothesized to play a role in maintaining the health of the animals. Indeed, among the microbes identified, are known short-chain fatty acid (e.g., butyric acid) producers (18, 19), which supports and expands upon those previously identified by Caballero et al. (4). Further, the use of Deblur to identify ESVs facilitates the temporal tracking of their relative abundances to inform selection of primary fecal samples that will provide the best probability (i.e., highest relative abundance) of culturing target taxa for downstream screening of probiotic potential. However, translating animal-derived observations from experimental animal models to human clinical situations remains challenging particularly where the key microbes are rodent-specific microbes. One solution may be to integrate metabolomics to reveal shared metabolic capacity among taxonomically divergent microbes. Our supervised classifying approaches suggests an altered metabolome composition during the late phase of VREfm challenge that may facilitate the apparent “suppression” of VREfm to levels below the limit of detection by culture. Despite the caveat of poor resolution in current databases to link metabolite features to associated chemical structures, microbe-metabolite vector analysis linked metabolite feature 6325 (*m/z* = 173.067 and RT = 18.392) to *Bacteroides* (Fig. 5). Our efforts toward manually identifying feature 6325 suggests a chemical formula of C_5_H_8_N_4_O_3_ and a structure likely to contain a N-acetylated hydroxyl group; a putative annotation (through *pubchem* search) is 3-hydroxy-4-(nitrosocyanamido) butyramide. Butyramide is the amide group of butyric acid, a short-chain fatty acid that has been shown to play a key role in colonization resistance against intestinal pathogens (20–23). Further research to comprehensively characterize interactions between microbe and metabolites will be critical to address the gaps in our understanding of the biochemical parameters that define interspecies microbiome interactions during antibiotic pretreatment and persistent infections.

The resolution of our results provides the basis in which to begin to identify nonantibiotic alternatives to engineer the gut microbiome through prebiotic interventions (e.g., butyric acid) and translating animal studies to human-relevant therapeutic applications by delineating taxonomically diverse microbes with shared metabolic capacity. Here, achieving integrative omics to link microbe-metabolite associations, our findings add support to the incorporation of microbiome profiling approaches into routine clinical microbiology, particularly in the context of monitoring the impacts of antibiotic use.

## MATERIALS AND METHODS

### Mouse gastrointestinal colonization model.

Six-week-old wild-type C57BL/6 male mice were used to establish an animal model of gastrointestinal colonization with VREfm. Mice were cohoused and had free access to food (ordinary chow) and water and had environmental enrichment (e.g., fun tunnels, chew blocks, and tissue paper). The light/dark cycle was 12-h light/12-h dark, and cages were changed weekly. Mice were pretreated with 0.5 g/liter ceftriaxone in drinking water for 2 days, followed by an antibiotic wean period of 24 h. Mice were then challenged with 1 × 10^6^ CFU VREfm ST796.

### Genomic DNA extraction and sequencing.

Whole-community genomic DNA (gDNA) was extracted from mouse fecal samples using the Qiagen PowerSoil DNA Extraction kit (formerly MoBio) following the manufacturer’s protocol. A preprocessing step of mechanical lysis was incorporated using a Bertin Technologies Precellysis 24 machine for one round of a 40-s cycle at 6,000 rpm. The V4 region of the bacterial 16S rRNA gene was amplified using small subunit 515 forward Golay-barcoded, and SSU806 reverse primers following the Earth Microbiome Project protocol (24), and sequenced using the Illumina MiSeq platform (V2, 300 cycles; Illumina Inc., San Diego, CA, USA). Further, primary derived data (e.g., BIOM tables) used to produce results can be found within QIITA study ID 11737.

### Amplicon 16S rRNA gene profiling analyses.

Sequence data were processed within the *QIITA* (v0.1.0) framework for quality control (25) (split libraries v. q2.1.9.1), demultiplexing, trimming sequence reads to a length of 150 nucleotides (nt), and picking suboperational taxonomic units (sOTUs) using *Deblur* (v1.1.0) to resolve single-nucleotide community sequencing patterns (i.e., feature identification of sOTUs [17]). The output BIOM files were further processed using *QIIME2* (v2019.7) for downstream statistical analyses (26). Alpha rarefaction curves were generated to determine whether each sample had been sequenced to saturation; the feature table was subsequently rarefied to 20,000 reads per sample. Taxonomy was assigned using the *sklearn* classifier (27) and *Greengenes 13.8 99% OTUs from 515F/806R region of sequences* classifier available from https://docs.qiime2.org/2018.4/data-resources/. Furthermore, relative abundances of each taxa were visualized as bar plots using the QIIME2 *taxa* plugin. A phylogenetic tree was constructed using fragment insertion (*QIIME fragment-insertion sepp* [28]) to guide phylogenetic-aware statistical analyses generated using the *QIIME2* plugin, *q2-diversity core-metrics-phylogenetic*; key metrics computed by default include both alpha-diversity (e.g., Shannon’s diversity index, Faith’s phylogenetic diversity, and evenness), and beta-diversity (e.g., Bray-Curtis distance and unweighted UniFrac distance) metrics. The unweighted UniFrac distance matrix (12) was used to compute first distances and calculate distances relative to day 0 as the baseline between sequential states (*QIIME longitudinal first-distances*); *ggplot2* (R v3.6.0; https://ggplot2.tidyverse.org) was used to visualize the distance scores as line plots. *Emperor* was used to visualize principal-coordinate analysis plots of unweighted UniFrac distances. Permutation-based statistical testing (PERMANOVA) on unweighted UniFrac distances was used to determine whether samples grouped by phase of experiment were significantly different from one another (*q2-beta-group-significance*). *Songbird* (https://github.com/mortonjt/songbird) was employed to determine the importance (i.e., fold change) of each sOTU in relation to a given metadata variable (e.g., VREfm colonization). Microbial features from all samples were split into training and test sets for supervised learning classifier analyses; 20% of input samples were allocated to train the Random Forest Classifier within QIIME2, the estimator method used for sample prediction. The different experimental phases were the response variables, while the 16S rRNA gene data were the features.

### Metabolite extraction and liquid chromatography-tandem mass spectrometry analysis.

Duplicate fecal samples, as outlined in Table 1, were processed for polar metabolite extraction and analysis (days 0, 5, 6, 7, 8, 9, and 14). Feces were metabolically arrested by immediate collection into dry ice, and stored at –80°C until further processing. Metabolite extraction from the fecal samples was undertaken by the addition of 500 μl per sample of methanol-water solution (3:1 [vol/vol]) containing 2 μM [^13^C]sorbitol and 8 μM [^13^C,^15^N]valine, and 2 μM [^13^C]leucine as internal standards. Fecal samples were homogenized at 1,200 rpm for 30 min in a thermomixer maintained at 4°C, mechanically disrupted, and incubated for a further 15 min in the thermomixer. Samples were randomized for metabolite extraction.

Metabolite analysis of the extracted samples, pooled biological quality control (PBQC) samples, and 13 mixtures of authentic standard mixes was performed by liquid chromatography-mass spectrometry (LC-MS) using hydrophilic interaction column (ZIC-pHILIC) and high-resolution Agilent 6545 series quadrupole time of flight mass spectrometry (QTOF MS) as described previously (29). PCoA of binary Jaccard distances of test, standard mixes, and PBQC samples are presented in Fig. S5 in the supplemental material. Ions were analyzed in positive mode with full scan range of 85 to 1,200 *m/z* and in data-dependent tandem MS mode to facilitate downstream metabolite identification.

10.1128/mSystems.00452-20.5 FIG S5 Emperor visualization displaying samples assayed for metabolomics. Principal coordinate analysis plot of distances between each sample based on their overlapping molecules as measured by binary Jaccard. Samples assayed include pooled biological quality control samples (purple) and samples of known standard metabolite mixtures (green). The general metabolome profile of test samples is retained. Download FIG S5, PDF file, 0.7 MB. Copyright © 2020 Mu et al. 2020 Mu et al. This content is distributed under the terms of the Creative Commons Attribution 4.0 International license.

10.1128/mSystems.00452-20.6 FIG S6 Paired feature abundance heatmaps. (A) Microbe abundances and (B) metabolite log centered abundances across each phase of the experiment. Heatmaps are aligned along the *x* axis (phase of experiment); the *y* axis displays microbe and metabolite abundances. Download FIG S6, PDF file, 0.4 MB. Copyright © 2020 Mu et al. 2020 Mu et al. This content is distributed under the terms of the Creative Commons Attribution 4.0 International license.

### Metabolomic analyses.

The data-dependent tandem MS data were processed using *MZmine2* (v2.39) (30) to generate tabular matrices of metabolite features (i.e., *m/z* and retention time [RT]). Masses were detected, and chromatograms built using *Peak Detection* methods within *MZmine2.* Chromatograms were deconvoluted, and isotopic peaks were grouped; grouped peaks were aligned using *join aligner*. *Peak list rows filter* method was applied to the aligned peaks, and gaps were filled using *peak finder*. The following MZmine2 settings were applied for spectral processing; MS1 mass detection, 1E3; MS2 mass detection, 1E2; time span, 0.02; minimum height, 3E3; *m/z* tolerance, 10 ppm; pairing *m/z* range for MS2, 0.1; RT range for MS2 scan, 2 min; minimum peak height, 7E3; peak duration, 0.02 to 5.00; baseline, 5E3;0.001, or maximum chance, 2; Join Aligner, 75% 25% ratio split; intensity tolerance, 10%; and *m/z* tolerance, 5 ppm. Feature finding produced a data matrix of MS1 features and associated peak areas. Feature-based molecular networking outputs (*quant.csv*) were generated from *MZmine2* using the *export to GNPS* module, which contains MS1 feature information and a corresponding *mgf* file containing MS2 information linked to MS1 features. Metabolomic features were further analyzed within the *Global Natural Products Social Networking* (*GNPS* v1.2.5 [13]) framework (University of California San Diego [UCSD], CA, USA). Tandem MS data were processed for identification, dereplication, and quantification, including spectral library searches. For example, MS2 spectra of the unknown metabolites are compared with a library of MS/MS spectra generated from structurally characterized metabolites. Further information on the GNPS workflow and molecular networking can be found in Wang et al. (13). Further, manual interpretation—including, for example, determining the molecular formula of the chemical in the neutral charge structure, determining the theoretical monoisotopic mass, and determining the likely adduct—of MS/MS data was applied to identify unknown features.

### Neural networking to predict microbe-metabolite interactions.

Microbiota and metabolome feature tables were analyzed using *MMVEC* (31) (https://github.com/biocore/mmvec) to identify microbe-metabolite interactions through their cooccurrence probabilities as predicted by neural networking. Conditional biplots were generated using *Emperor*. Further, microbe abundances and metabolite log centered abundance heatmaps were generated using primary derived data from multinomial regression analyses using *Songbird* (https://github.com/mortonjt/songbird).

### Data availability.

Amplicon 16S rRNA gene sequencing and metabolomic data for this study were deposited in publicly available databases. Raw sequence data are available through the European Nucleotide Archive, accession number PRJEB39605. Raw spectral data for metabolomics are available through https://massive.ucsd.edu, accession number MSV000085847.
