# Supplementary figures and images for: Microbe-Metabolite Associations Linked to the Rebounding Murine Gut Microbiome Postcolonization with Vancomycin-Resistant Enterococcus faecium
Source: mSystems. 2020 Aug 18;5(4):e00452-20. doi: 10.1128/mSystems.00452-20 (PMC7438022; doi:10.1128/mSystems.00452-20)

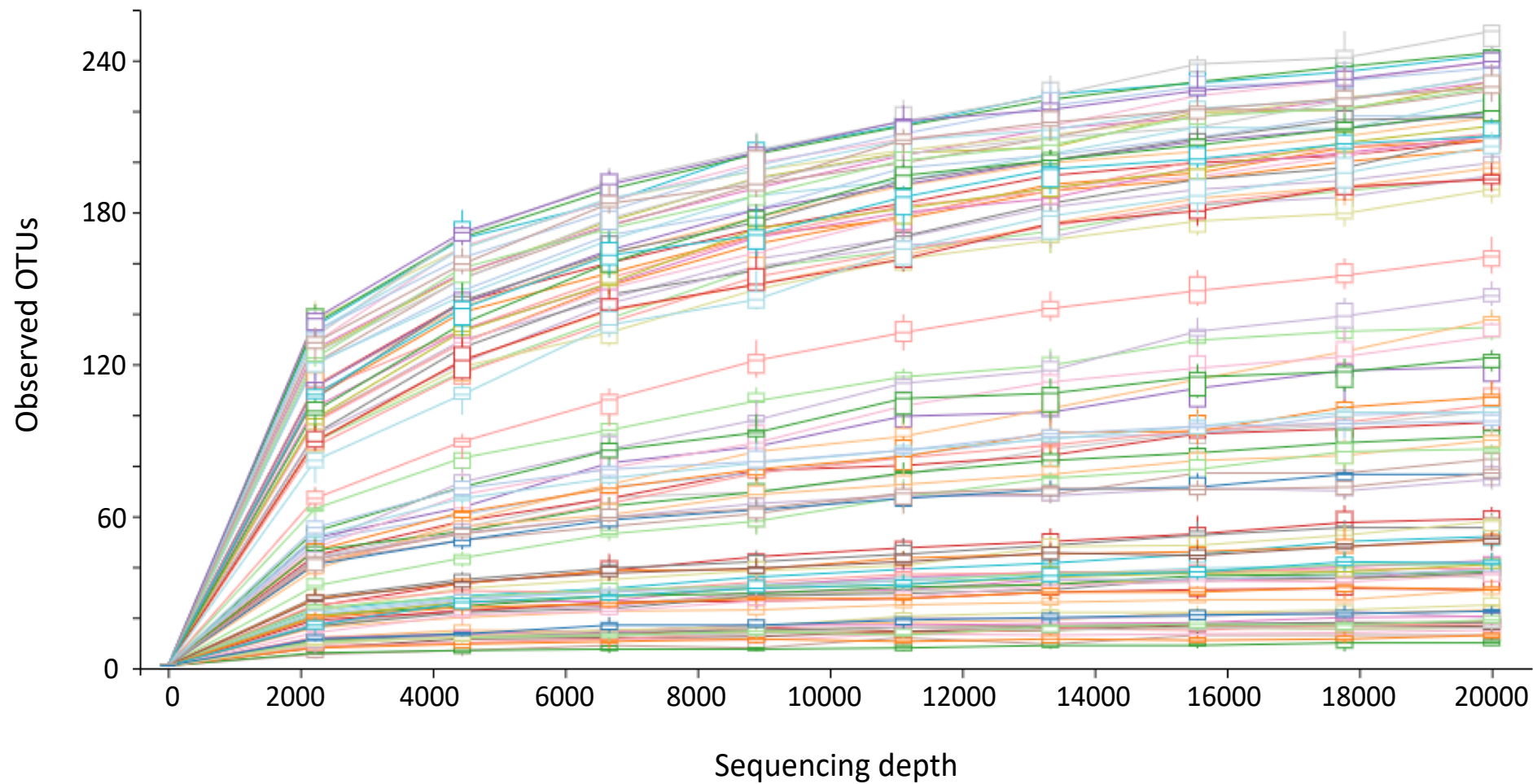

Supplement: FIG S1 [file mSystems.00452-20-sf001.pdf]

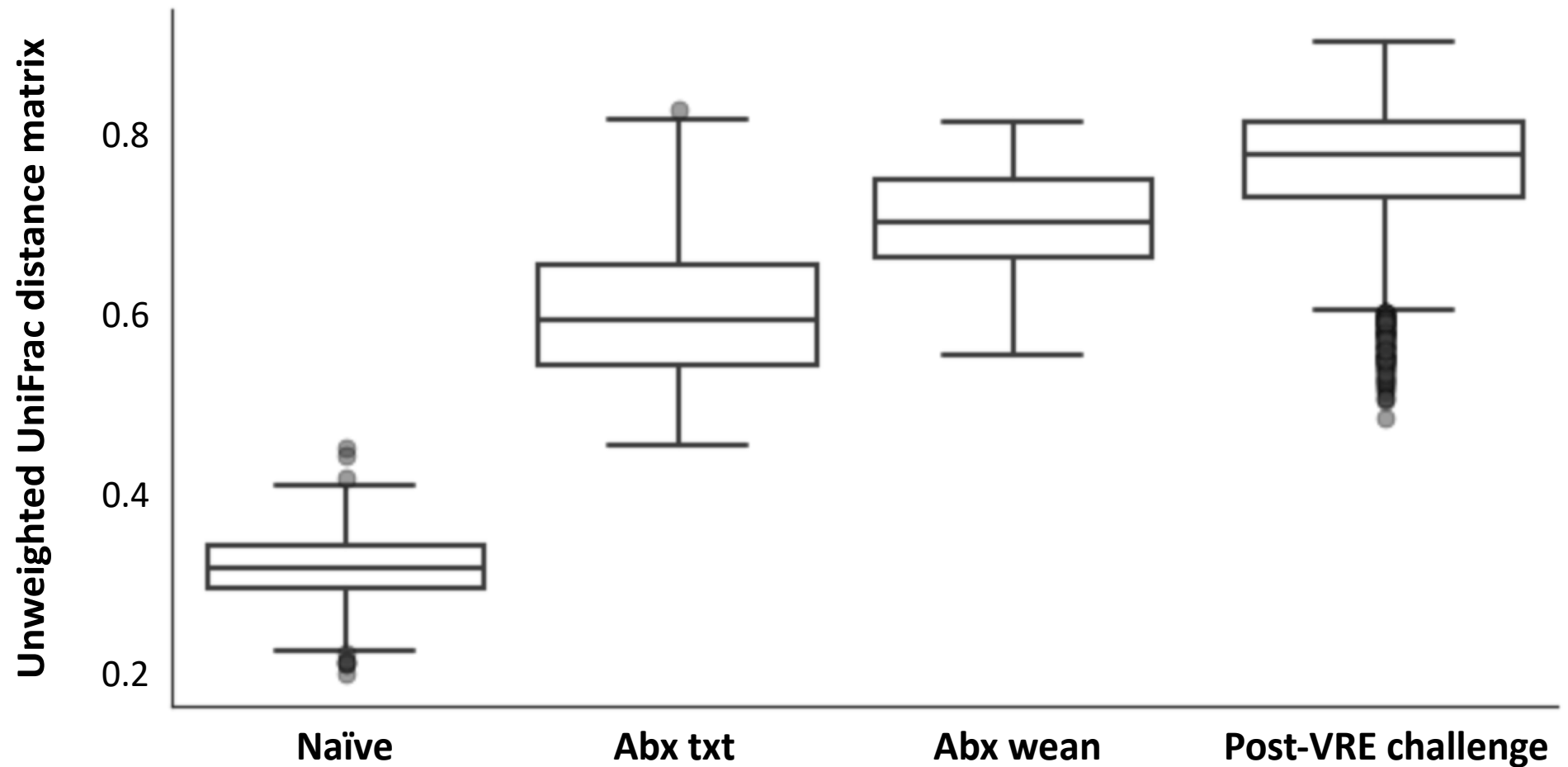

Supplement: FIG S2 [file mSystems.00452-20-sf002.pdf]

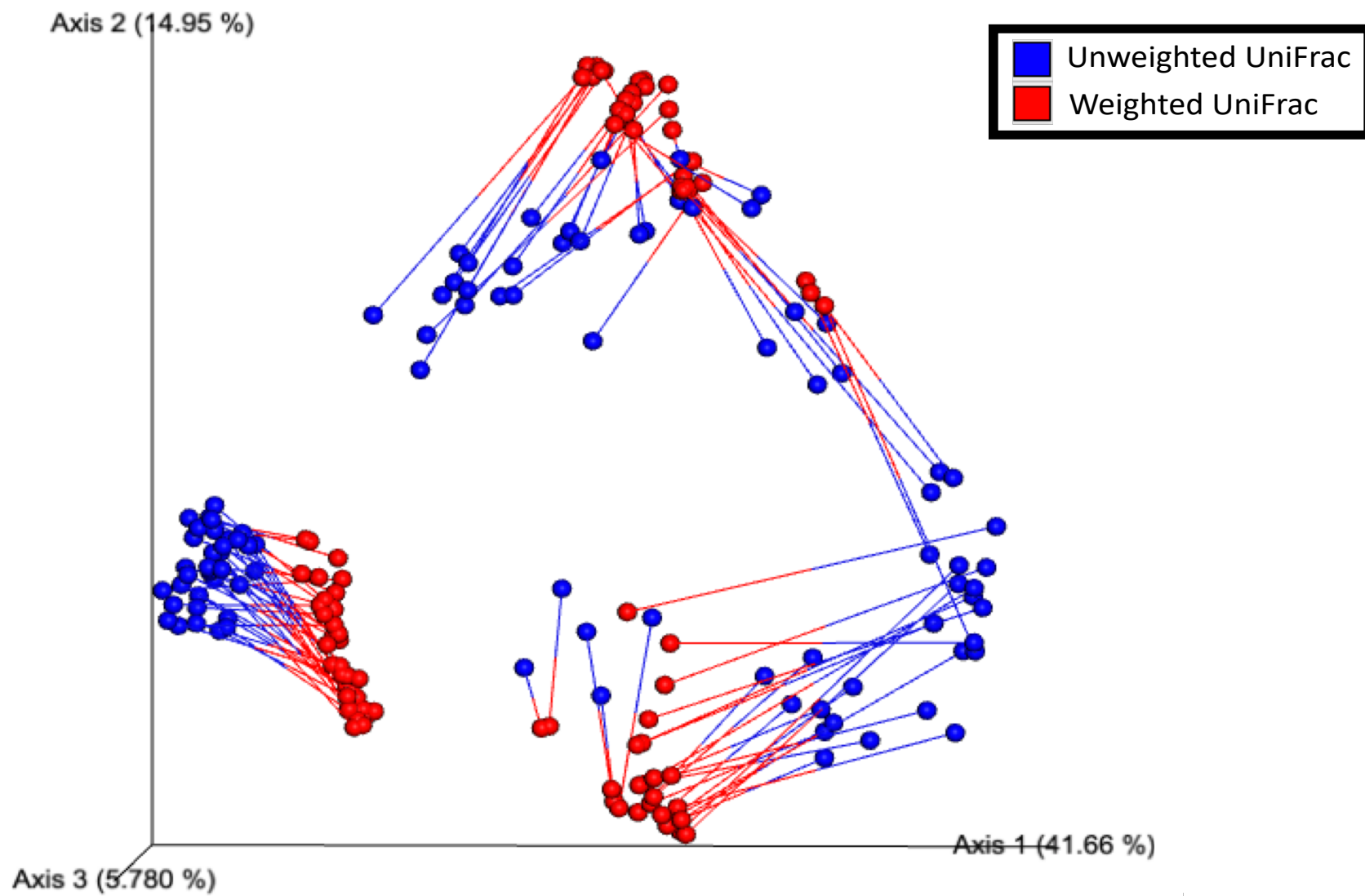

Supplement: FIG S3 [file mSystems.00452-20-sf003.pdf]

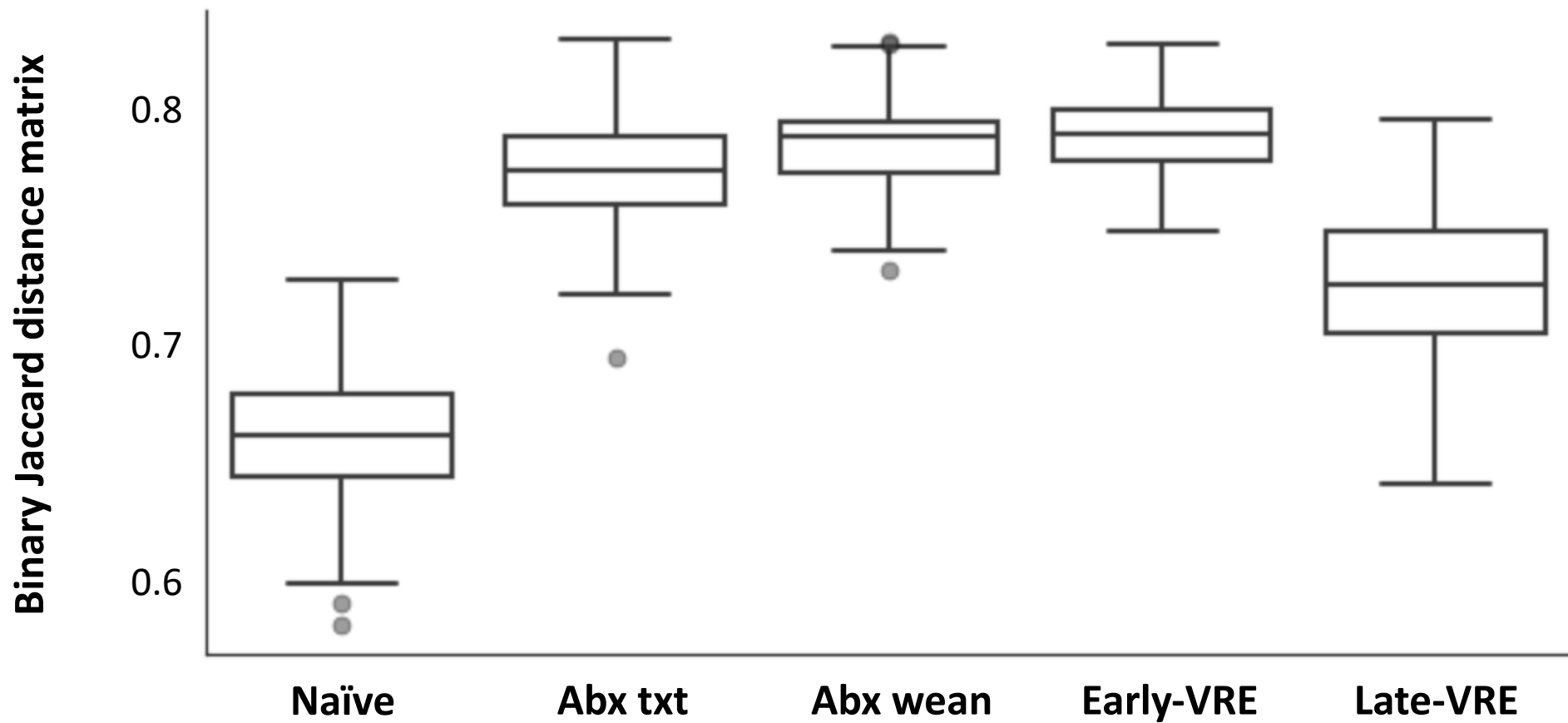

Supplement: FIG S4 [file mSystems.00452-20-sf004.pdf]

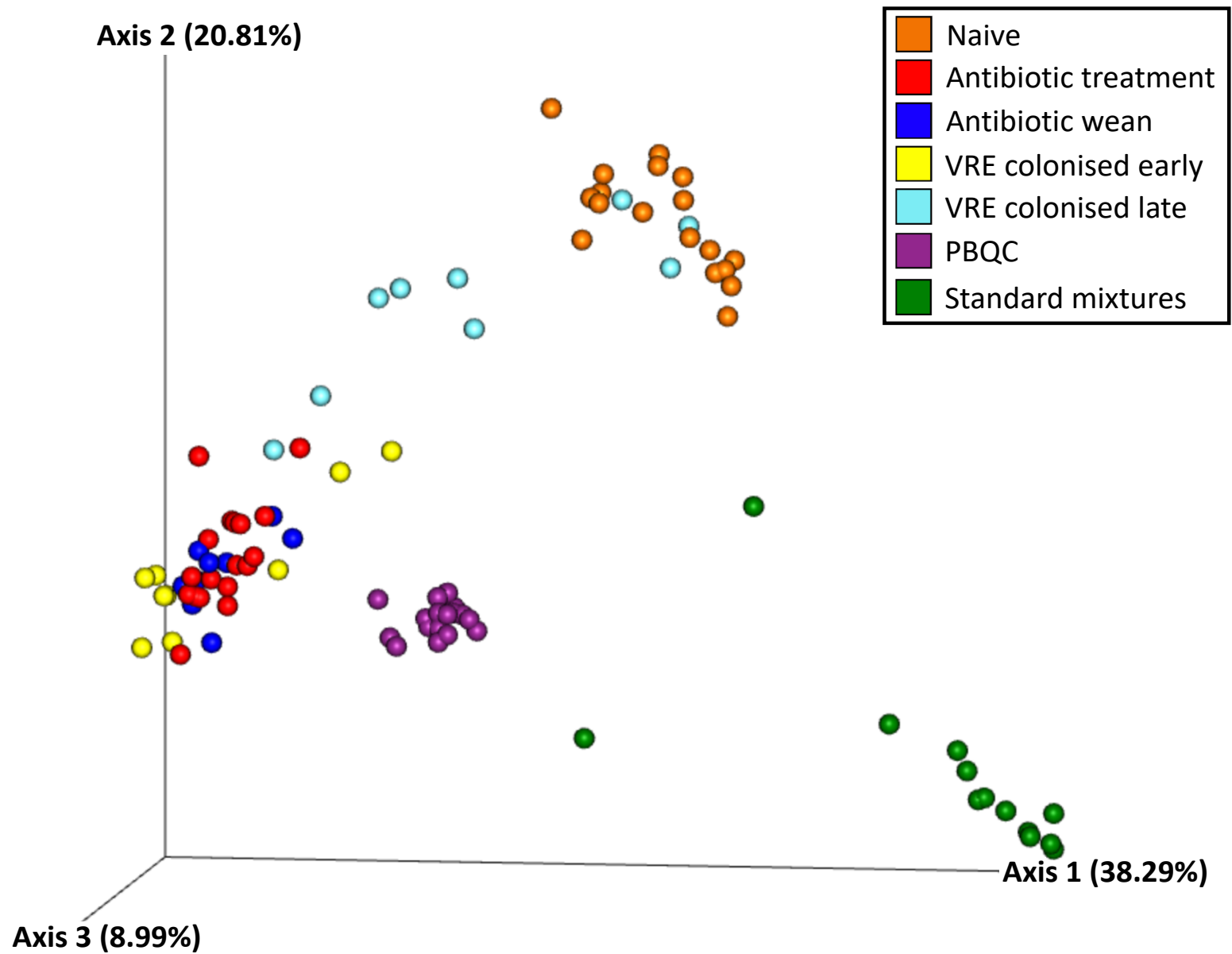

Supplement: FIG S5 [file mSystems.00452-20-sf005.pdf]

**A**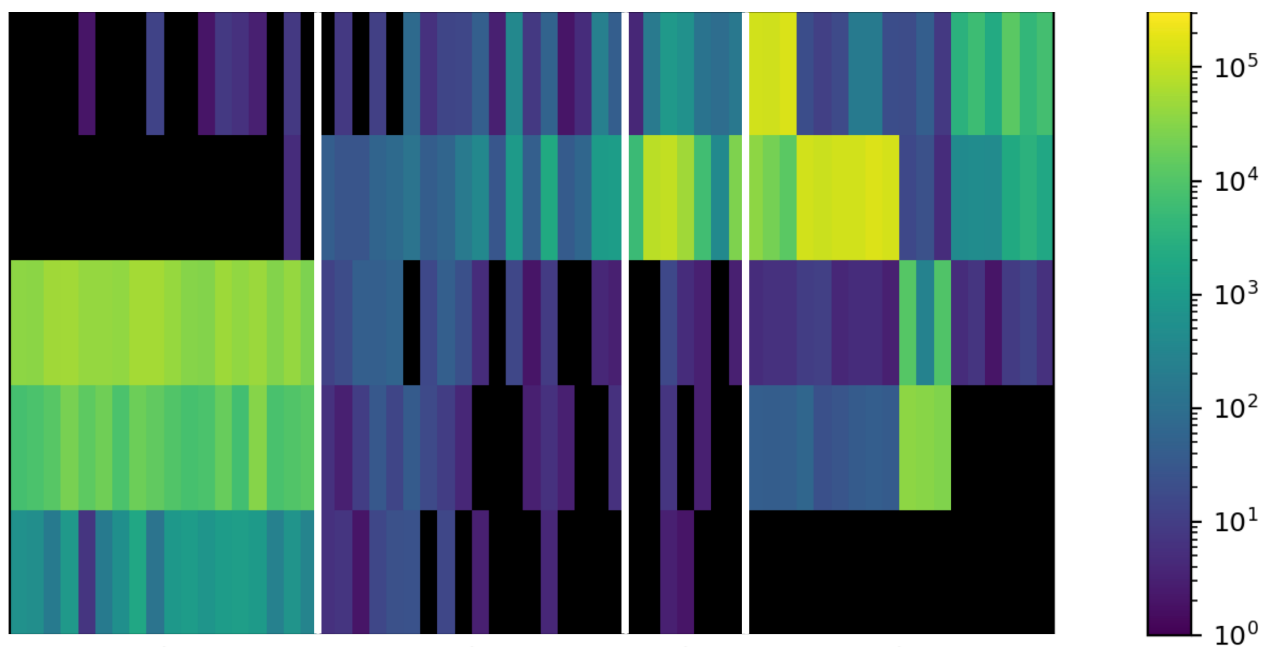**B**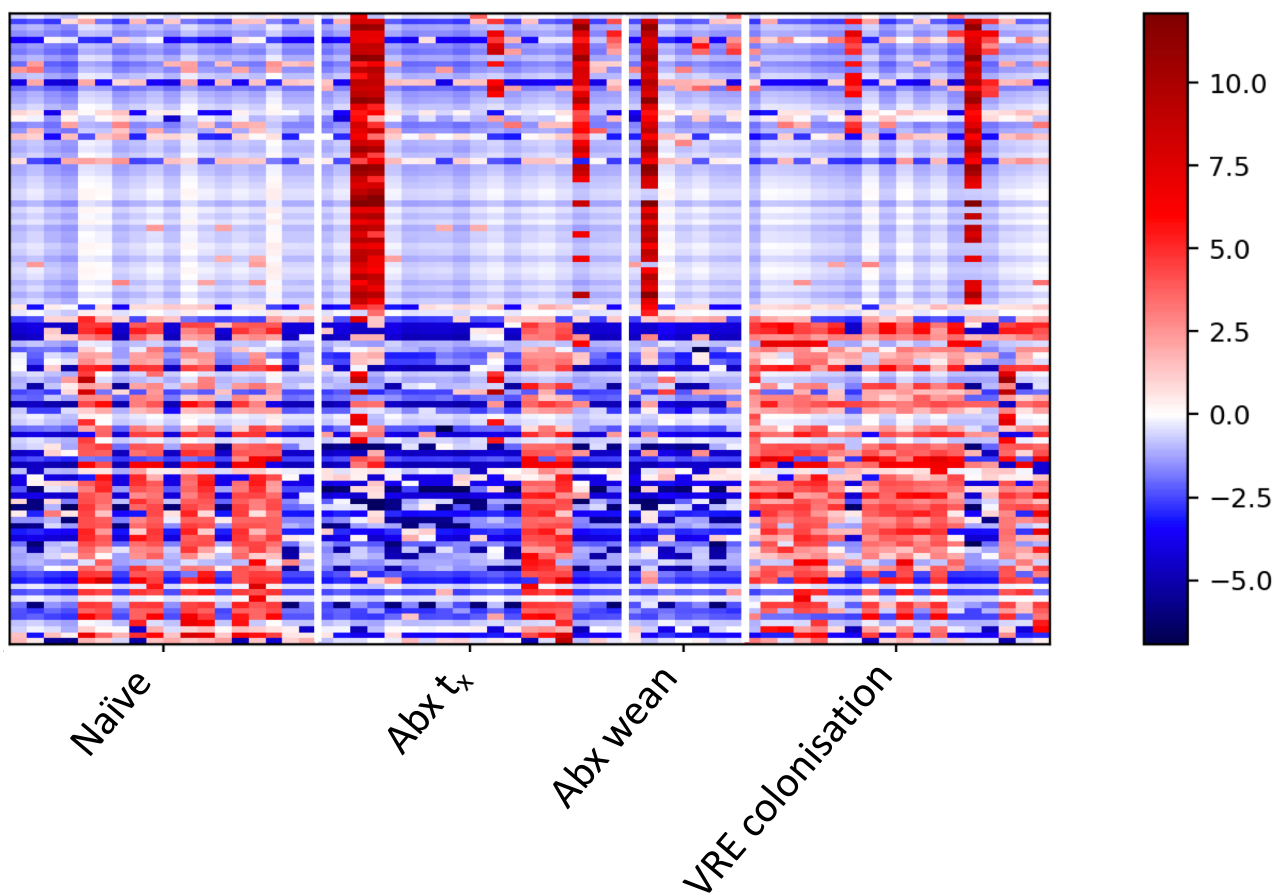

Supplement: FIG S6 [file mSystems.00452-20-sf006.pdf]
